# Enhancing accessibility: a multi-level platform for visual question answering in diabetic retinopathy for individuals with disabilities

**DOI:** 10.3389/frai.2025.1646176

**Published:** 2025-11-03

**Authors:** Sarah Alotaibi, Suheer Al-Hadhrami, Saad Al-Ahmadi

**Affiliations:** ^1^Department of Computer Science, College of Computer and Information Sciences, King Saud University, Riyadh, Saudi Arabia; ^2^Computer Engineering Department, College of Engineering, Hadhramout University, Al Mukalla, Yemen

**Keywords:** disability-aware VQA, ELECTRA, Med-VQA, medical visual question answering, multi-level VQA, question answering, SWIN, vision-language models

## Abstract

Individuals with visual disabilities possess impairments that affect their ability to perceive visual information, ranging from partial to complete vision loss. Visual disabilities affect about 2.2 billion people globally. In this paper, we introduce a new multi-level Visual Questioning Answering (VQA) framework for visually disabled people that leverages the strengths of various VQA models of the multi-level components to enhance system performance. The model relies on a bi-level architecture that employs two distinct layers. In the first level, the model classifies the question type. This classification guides the visual question to the appropriate component model in the second level. This bi-level architecture incorporates a switch function that enables the system to select the optimal VQA model for each specific question, hence enhancing overall accuracy. The experimental findings indicate that the multi-level VQA technique is significantly effective. The bi-level VQA model enhances the overall accuracy over the state-of-the-art from 87.41% to 88.41%. This finding suggests the use of multiple levels with different models can boost the VQA systems' performance. This research presents a promising direction for developing advanced, multi-level VQA systems. Future work may explore optimizing and experimenting with various model levels to enhance performance further.

## 1 Introduction

Visual disabilities affect millions of people worldwide, posing a major global concern. These impairments severely restrict access to visual information and limit participation in many daily activities (Gurari et al., 2018). The World Health Organization (WHO) reports that more than 2.2 billion individuals worldwide experience some form of visual impairment or blindness, with many cases arising from preventable or treatable conditions such as diabetic retinopathy (DR) (Organization, 2019).

The standard practice for diagnosing and assessing DR involves ophthalmologists manually analyzing fundus images to determine disease severity. However, this method is time-intensive, error-prone, and highly subjective. The global shortage of ophthalmologists further exacerbates these challenges (Abràmoff et al., 2018).

These limitations hinder timely and accurate diagnosis, especially as the prevalence of DR continues to rise. Recent deep learning developments show promise in automating DR detection and grading, offering potential solutions to these challenges. However, these methods face practical limitations, including data scarcity, difficulty generalizing to real-world scenarios, and suboptimal performance in handling complex diagnostic questions (Jagan Mohan et al., 2022). Advancements in assistive technologies powered by artificial intelligence (AI) promise to transform lives, enhance independence, and elevate the quality of life for these individuals (de Freitas et al., 2022). Recent deep learning developments To bridge this gap, Visual Question Answering (VQA) has emerged as a promising development capable of extracting meaningful insights by answering user-defined questions based on image content (Lin et al., 2023). In the medical domain, Medical Visual Question Answering (Med-VQA) has recently become a potential solution (Lin et al., 2023). Med-VQA combines advancements from Computer Vision (CV) and Natural Language Processing (NLP) to answer complex medical questions using images like fundus images, CT scans, and X-rays (Lin et al., 2023; Gu et al., 2024).

Integrating text with image data, Med-VQA offers several advantages. It facilitates expedited and accurate diagnoses for physicians. It also alleviates their workload by delivering immediate responses to common inquiries and provides medical students with a valuable study resource.

Additionally, Med-VQA empowers patients by providing access to information regarding their ailments using straightforward question-and-answer interfaces (Al-Hadhrami et al., 2023). The Med-VQA area is nascent and has numerous constraints, notably the lack of high-quality labeled data (Gu et al., 2024). Presently accessible datasets such as VQA-RAD (Zhu et al., 2016), SLAKE (Liu et al., 2021), VQA-Med (Abacha et al., 2019, 2020), and DME (Tascon-Morales et al., 2022) serve as foundational references. Nonetheless, numerous efforts are inadequate due to insufficient question diversity, limited data volumes, and, in certain instances, the lack of clinical validation, hindering the development of robust and generalizable models.

In the context of available Med-VQA datasets, this study tackles the challenge of the data limitation by fine-tuning models on comprehensive datasets that encompass various types and modalities of questions (Zhang et al., 2023). By utilizing all available data and focusing on specific question types or classes during the fine-tuning process, the proposed methodology mitigates issues related to model generalization and overfitting. The hierarchical model structure introduced in this research outperforms conventional methods by categorizing visual question types according to image, text, or combined image-text. This distinctive classification methodology that highlights the image and text modalities differs this work from the existing literature and introduces a new direction for improved VQA performance.

In the context of VQA, Al-Hadhrami et al. (2023) demonstrated that models fine-tuned using various hyperparameters perform best for different question types or response classes. This finding highlights the importance of models being designed for given question classes, the next fundamental step toward the enhancement of the effectiveness and efficiency of the models for VQA. Based on this finding, this work highlights the importance for models being flexible and adaptive for handling multiple question types dynamically, eventually enhancing the performance and delivering more accurate responses for real-world applications.

State-of-the-art (SOTA) methods often incorporate several advanced techniques. These include joint embedding (Ren et al., 2015; Antol et al., 2015; Malinowski et al., 2015), attention mechanisms (Jiang et al., 2015; Chen et al., 2015; Ilievski et al., 2016; Andreas et al., 2016b; Song et al., 2022), compositional reasoning (Andreas et al., 2016a,b; Xiong et al., 2016; Kumar et al., 2016; Noh and Han, 2016; Gao et al., 2019), and knowledge-enhanced approaches (Wang et al., 2015, 2017; Wu et al., 2016; Zhu et al., 2016).

More recently, most models have attempted to employ attention mechanisms for mapping text and image features together (Peng et al., 2018; Lu et al., 2016; Chen et al., 2015; Shi et al., 2018). Moreover, pre-trained visual-language (V + L) models such as visualBERT (Li et al., 2019a), UNITER (Chen et al., 2020b), VilBERT (Lu et al., 2019), and CLIP (Radford et al., 2021) demonstrated their ability for increased performance. Additionally, researchers also began using image captioning to provide models with increased text context for understanding complex medical queries (Cong et al., 2022). However, despite these advancements, the available models fail to easily deal with the diversity of the type of questions encountered during real-life medical scenarios. This lack of adaptability limits their utility and points toward the need for novel approaches for the unique Med-VQA concerns. This paper addresses the limitations of existing Med-VQA approaches by introducing a new architecture that improves flexibility and accuracy in answering medical questions. Unlike conventional models, our approach hierarchically categorizes questions based on their dependence on image, text, or mixed modalities. This classification allows for the fine-tuning of models for each form of the question independently, eliminating the generalization and overfitting issues. Besides, by using the switch function for adaptive best-fitting model selection for each form of the question, the solution is extremely flexible and adjustable, providing much improved performance and accuracy.

The key contributions of this study are listed as follows:

- This study introduces a novel multi-level VQA framework designed to handle diverse medical question types by categorizing them hierarchically based on their reliance on image, text, or combined modalities.
- The proposed VQA system employs a bi-level architecture where the first-level classifies the input question type. The second level utilizes specialized component models for each question type, improving accuracy by dynamically selecting the most suitable model using a switch function.
- The bi-level model is constructed using components selected from the best-performing state-of-the-art models on diabetic retinopathy. This design demonstrates how the proposed approach can enhance their performance within a unified framework. Those models are the ELECTRA-SWIN and two GS-ELECTRA-SWIN models with different hyper-parameters.

The subsequent sections of the paper are structured as follows: Section 2 presents the existing literature and methodologies for Med-VQA, highlighting current limitations and research gaps. Section 3 details the proposed hierarchical Med-VQA framework, encompassing its architecture and implementation. Section 4 presents the experimental results and discusses the performance improvements achieved by the proposed method. Finally, Section 5 presents with principal findings, implications, and recommendations for future research endeavors.

## 2 Related works

VQA systems generally consist of four essential elements: vision featurization, text featurization, fusion models, and answer classification or generation. These elements collectively enable the effective processing of image-based queries.

### 2.1 Vision Featurization

In the domain of VQA, vision featurization is a fundamental component of the multimodal architecture. Its primary role is to extract essential visual information from images. Representing an image as a numerical vector–known as image featurization—involves applying various techniques. These techniques include the scale-invariant feature transform (SIFT) (Lowe, 1999), simple RGB vectors, histogram of oriented gradients (HOG) (Dalal and Triggs, 2005), Haar transform (Lienhart and Maydt, 2002), and deep learning methodologies.

Deep learning approaches, particularly Convolutional Neural Networks (CNNs), play a pivotal role in visual feature extraction by leveraging neural networks to learn essential visual features. Deep learning can involve training models from scratch, which requires large datasets. Alternatively, transfer learning techniques yield strong performance even with limited data. Given the constraints of medical VQA datasets, researchers often resort to leveraging pre-trained models to enhance performance.

Widely used pre-trained models include AlexNet (Krizhevsky et al., 2017), VGGNet (Simonyan and Zisserman, 2015; Zhang et al., 2019; Abacha et al., 2018; Verma and Ramachandran, 2020a; Bounaama and Abderrahim, 2019), GoogLeNet (Szegedy et al., 2015), ResNet (He et al., 2016; Fukui et al., 2016; Kim et al., 2017; Ben-Younes et al., 2017; Huang et al., 2023; Tascon-Morales et al., 2022, 2023; Haridas et al., 2022), and DenseNet-121 (Kovaleva et al., 2020). These architectures have shown strong effectiveness in extracting image features.

In addition, ensemble models—combinations of multiple neural networks—have gained traction in vision feature extraction within VQA systems. By aggregating outputs, ensembles can outperform individual models. This potential has motivated researchers to explore their utility in enhancing vision feature extraction (Liao et al., 2020; Do et al., 2021; Gong et al., 2021; Wang et al., 2022a,b).

### 2.2 Text featurization in visual question answering

Text featurization, Like vision featurization, is crucial in converting questions into numeric vectors and facilitating mathematical computations in VQA systems. The selection of an appropriate text embedding method often involves an iterative process (Manmadhan and Kovoor, 2020). Various text embedding techniques employed in SOTA models significantly influence the multi-modal nature of VQA systems.

Among the prevalent text embedding methods used in question modeling, Long Short-Term Memory (LSTM) (He et al., 2020b; Kovaleva et al., 2020; He et al., 2020a; Tascon-Morales et al., 2022, 2023; Wang et al., 2022a,b), Gated Recurrent Units (GRU) (He et al., 2020b,a), Recurrent Neural Networks (RNNs) (Allaouzi et al., 2018; Abacha et al., 2018; Zhou et al., 2018b; Talafha and Al-Ayyoub, 2018), Faster-RNN (He et al., 2020b,a), and the encoder-decoder approach (Vu et al., 2020; Fukui et al., 2016; Kim et al., 2017; Ben-Younes et al., 2017; Verma and Ramachandran, 2020b; Kiros et al., 2015) are widely utilized.

Moreover, pre-trained models like Generalized Auto-regressive Pre-training for Language Understanding (XLNet) (Yang et al., 2019) and Bidirectional Encoder Representations from Transformers (BERT) (Devlin et al., 2019; Verma and Ramachandran, 2020b; Huang et al., 2023; Haridas et al., 2022) have gained prominence in text featurization within VQA frameworks. Notably, specific models opt to bypass explicit text featurization, treating the problem as an image classification task (Gong et al., 2021; Eslami et al., 2021; Schilling et al., 2021). This enhanced description of text featurization in VQA systems emphasizes the diverse range of methods and pre-trained models used to transform textual queries into numerical representations, thereby enhancing the model's overall performance and multimodal capabilities.

### 2.3 Fusion in visual question answering systems

The fusion step in VQA systems involves the integration of independently extracted text and image features. This fusion process serves as a pivotal stage in VQA pipelines. Manmadhan and Kovoor (2020) have categorized fusion into three main categories: baseline fusion models, joint attention models, and end-to-end neural network models. Baseline fusion models encompass a variety of fusion techniques, such as element-wise addition (Antol et al., 2015), element-wise multiplication, and concatenation (Zhou et al., 2018a). They also include combinations of these methods (Malinowski et al., 2017) and hybrid approaches involving polynomial functions.

End-to-end neural network models are instrumental in seamlessly merging image and text features. Noteworthy methods include neural module networks (NMNs) (Andreas et al., 2016b), multi-modal approaches like MCB (Fukui et al., 2016), and dynamic parameter prediction networks (DPPNs) (Noh et al., 2016). Other approaches include multi-modal residual networks (MRNs) (Kim et al., 2016), cross-modal multistep fusion (CMF) networks (Mingrui et al., 2018), and basic MCB models enhanced with deep attention neural tensor network (DA-NTN) modules (Bai et al., 2018). Additional methods employ MLPs (Narasimhan and Schwing, 2018) and encoder-decoder techniques (Chen et al., 2020a; Li et al., 2019b).

Joint attention models include the word-to-region attention network (WRAN) (Peng et al., 2018), co-attention mechanisms (Lu et al., 2016), question-guided attention maps (QAM) (Chen et al., 2015), and question type-guided attention (QTA) (Shi et al., 2018). These approaches are designed to capture nuanced semantic relationships between text and image attentions (Manmadhan and Kovoor, 2020).

In addition to traditional neural network methods like LSTM and encoder-decoder architectures, Verma and Ramachandran (Verma and Ramachandran, 2020b) have introduced a multi-model approach incorporating encoder-decoder, LSTM, and GloVe embeddings. Moreover, integrating vision-language pre-trained models, as seen in Haridas et al. (2022), further enriches the fusion process within VQA systems. Recent improvements in VQA show that most methods integrate vision and text processing to enhance accuracy. Vision featurization now relies on CNNs for detailed feature extraction, while text featurization employs models such as LSTMs and BERT for efficient question encoding. Advanced fusion techniques, especially attention-based networks, refine image-text alignment and push VQA toward higher levels of cross-modal understanding and performance. Recent work by Tascon-Morales et al. (2023) benchmarked transformer-based VQA models on datasets like VQA-RAD and PathVQA. This revealed problems with dataset bias and generalization. Other models, such as ViLT, VisualBERT, and GLoRIA, perform well due to vision-language pretraining and attention-based fusion. Unlike these flat architectures, our approach introduces a bi-level structure with question-type routing to improve specialization and robustness.

## 3 Proposed method

A multi-level VQA system is a VQA that has multiple levels, each with several VQAs to handle particular questions or answers. This section highlights the methodology for the multi-level VQA system, encompassing problem specification, an outline of the proposed approach, a description of its elements, and subsequent model training strategies.

This section delineates the methodology for the multi-level VQA system, including problem specification, an overview of the proposed approach, a description of its components, and subsequent model training procedures.

### 3.1 The proposed method overview

**Figure 1:**
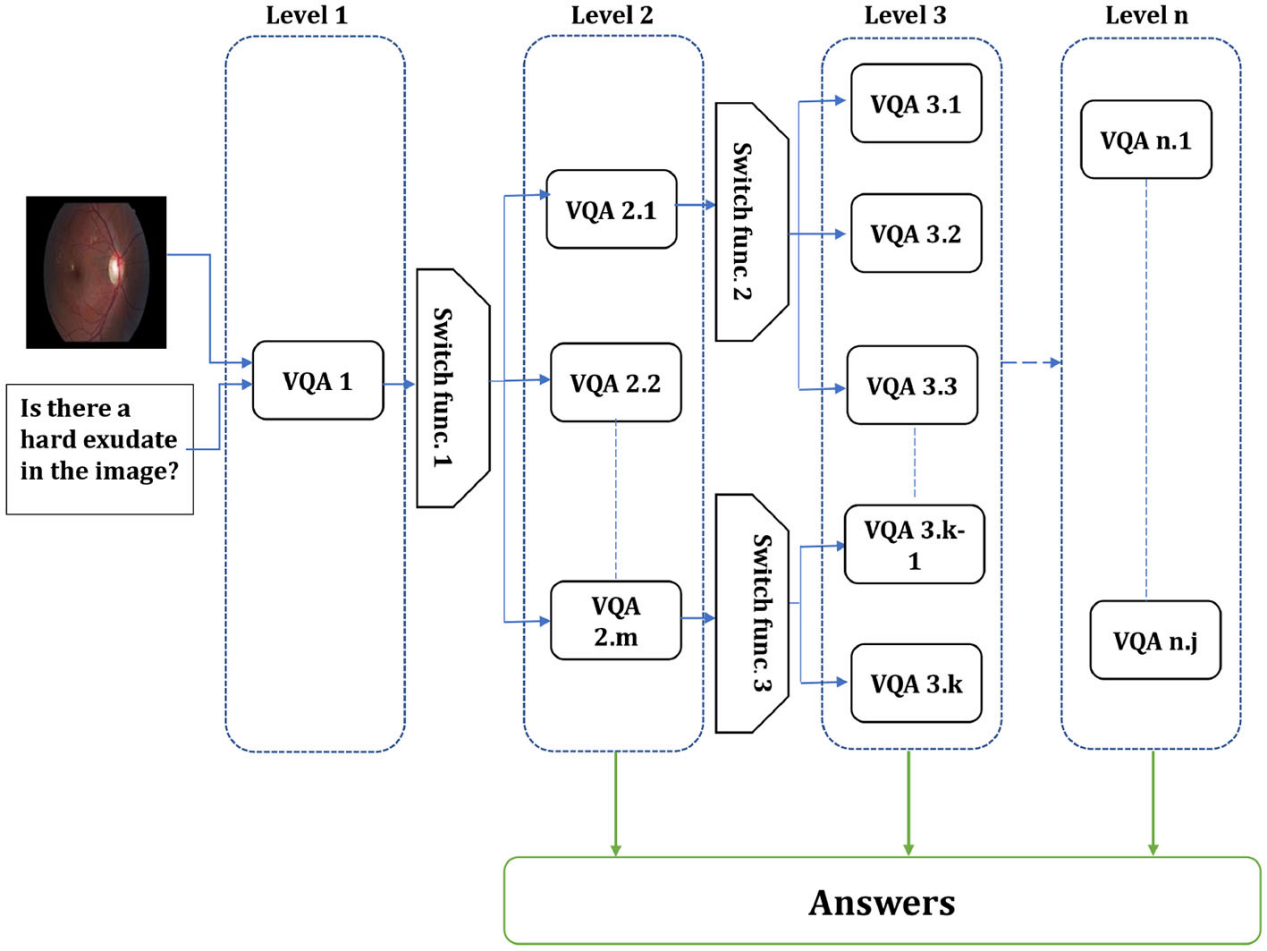
Overall structure of the multi-level VQA framework. The system is composed of n levels, each containing multiple models. The input image and question are first processed at first-level, and the switch function routes them to the appropriate model in the next level. The final answer is produced by one of the models, depending on the routing decisions across levels.

From the VQA models presented by Al-Hadhrami et al. (2023), we found that the models with various hyper-parameters outperform each other in different question types or particular answers. Therefore, designing models focusing on appropriate question types or classes has become increasingly crucial to enhancing the performance and effectiveness of VQA models. The flexibility of this approach allows for the customization of models to fit specific question types, leading to improved performance and more accurate answers. The existing Med-VQA datasets, several methods exist to handle the data and fine-tune the models on these datasets. For example, the dataset with multi-modality images and the dataset with different question types. Since limited data is one gap in the Med-VQA, splitting the data into sub-data can affect the model generalization and lead to overfitting. Therefore, using all data to fine-tune the model by focusing only on particular question types or classes could help to overcome those issues. In SOTA, a hierarchical model is proposed by splitting the data based on the image modality using image modality recognition or question type, such as open and closed, based on text only. Visual question types can be classified based on image, text, or both. The first two question type classifications are used in the literature, while we proposed utilizing the last method. In this paper, the multi-level VQA model is a hierarchical model composed of multiple levels of VQAs, each addressing specific aspects of the question. The predicted answer could be gained from the different model levels or the last one. For instance, one level may handle image-related inquiries, the next level focuses on question types, and the final level addresses the primary visual question. The investigation of the multi-level VQA model aims to improve overall performance. This study employs a Bi-level VQA model. Figure 1 shows the overall multi-level VQA method structure. Each level contains two or more VQA except the first-level, which includes one or more VQA. In this study, the first-level only has a single VQA. A single or multiple switch function separates the levels from each other. Algorithm 1 shows the procedure of the multi-level VQA framework.

**Algorithm 1:** Multilevel VQA system.

**Require:** Image *I*, Question *Q*, Set of Levels *L* = {*L*_1_, *L*_2_, …, *L*_*n*_}
**Ensure:** Answer *A*
1: Initialize *current*_*level*←1
2: *i*←1 {*i* is the level number}
3: *j*←1 {*j* is the model number}
4: *a*_*ij*_←*model*(*I, Q*) {*a*_*ij*_ is the answer of the model *j* in the level *i*}
5: **while** *current*_*level* ≤ *n* **do**
6: *models*←*L*_*Next*_*level*_ {Retrieve models in current level}
7: *selected*_*model*_*ij*_←SwitchFunction(*I, Q, models, a*_*ij*_) {Select the most suitable model}
8: *a*_*ij*_←*selected*_*model*(*I, Q*) {Answer from selected model}
9: {Intermediate answer is used only by switch function and not passed to next level}
10: **if** *a*_*ij*_ is final answer **then**
11: *A*←*a*_*ij*_
12: **return** *A*
13: **end if**
14: *current*_*level*←*current*_*level*+1
15: **end while**
16: *A*←*a*_*ij*_
17: **return** A

#### 3.1.1 Problem specification and formulation

Med-VQA attempts to accurately predict the correct answers based on a combination of medical images and text questions. The task requires generating a brief and accurate textual answer from the input pair comprising a medical image *I* and a question *Q*. This process is mathematically expressed as in Equation 1:


(1) $$\begin{array}{l} A=VQA\left(I,Q,\Theta \right) \end{array}$$

where *I* is the input image, *Q* is the question, *A* is the predicted answer, and Θ represents the model parameters.

In multi-level VQA (*MVQA*), both *I* and *Q* are processed by several models *M*_*k*_, where *k* < = *n*, *n* is the levels number. Each level *i* has *j* models, where *j*>0. Therefore, *M*_*ij*_ denotes to the model *j* in the level *i*. Those levels are separated by switch function *S*_*i*_, where *i* is the level that precedes it. So, the intermediate answers at each level given by in Equation (2):


(2) $$\begin{array}{l} a_{ij}=M_{ij}\left(I,Q\right) \end{array}$$

where *a*_*ij*_ is the answer of the models *j* in the level *i*.

and the decision to proceed to the next level is given by in Equation (3):


(3) $$D_{i-1}=\{\begin{array}{ll} 0 & \text{if no extra level and final answer is detected}\\ 1 & \text{if routing to the next }M_{\left(ij\right)} \end{array}$$

The final answer is given by in Equation (4):


(4) $$\begin{array}{l} A=M_{ij}\left(I,Q\right) \end{array}$$

where *D*_*i*−1_ = 0.

#### 3.1.2 Bi-Level VQA

**Figure 2:**
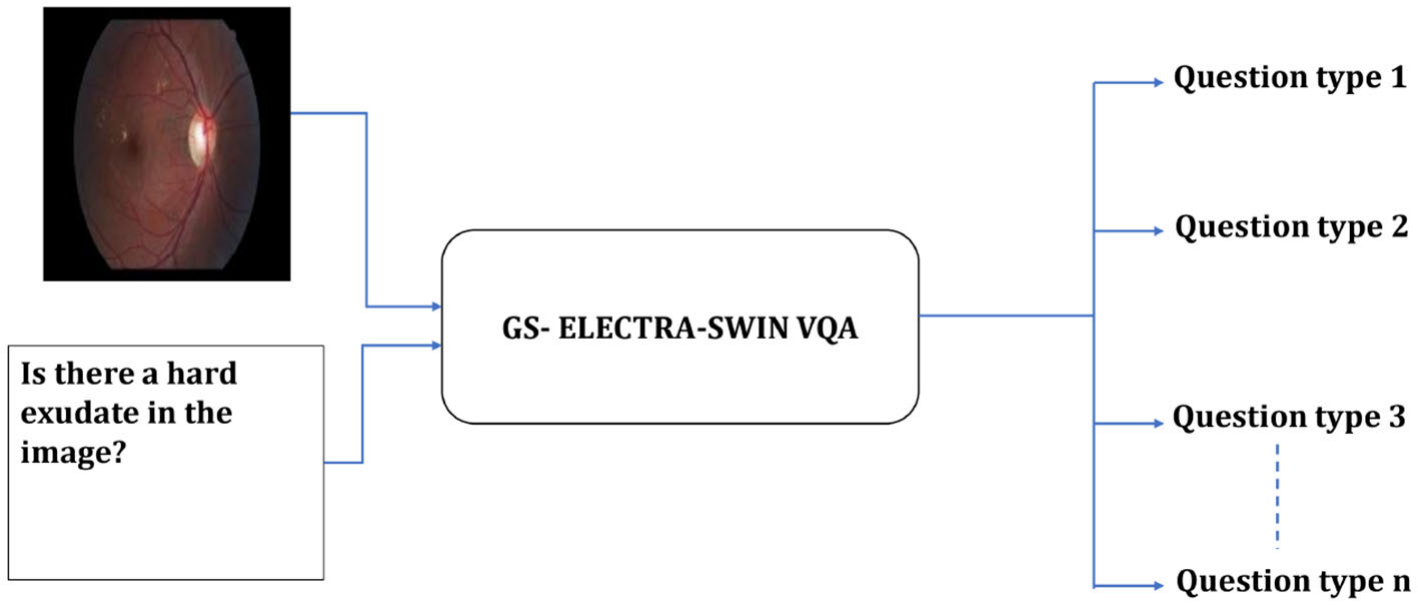
Overall structure of the first-level in the multi-level VQA framework. The model predicts the question type but does not provide the final answer.

Our proposed model is designed to enhance accuracy and efficiency by leveraging a hierarchical structure consisting of two distinct levels. The first-level serves as a classification system, which identifies the type of input question and produces specific information to guide subsequent processing. A switch function uses this information to route the visual question to the suitable VQA model in the second level to predict the answer. The first-level employs the GS-ELECTRA-SWIN VQA model as shown in Figure 2, known for its efficiency in question types classification, as discussed by Al-Hadhrami et al. (2023).

**Figure 3:**
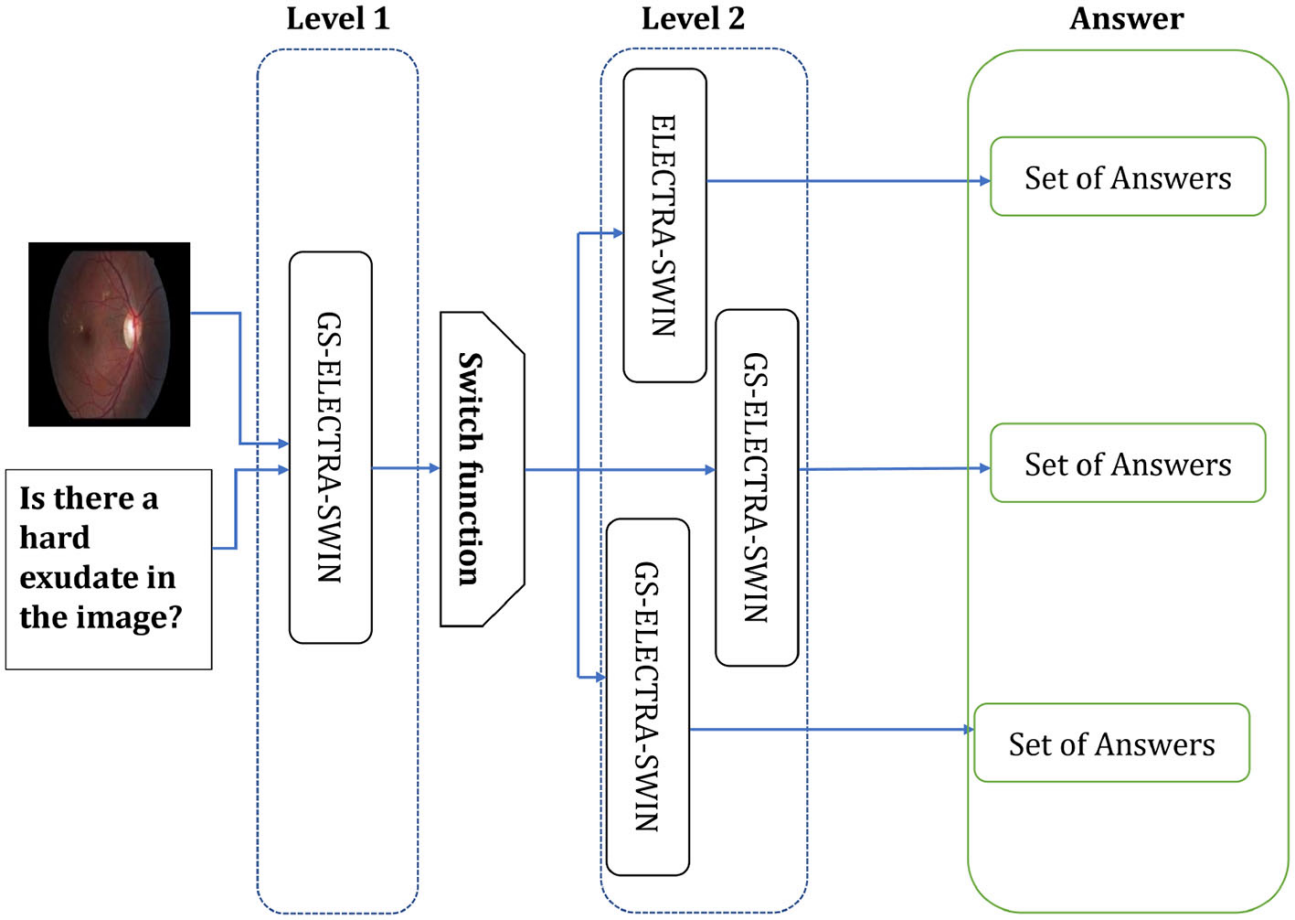
The Overall second-level structure of the multi-level VQA technique. Second-level structure: the first-level predicts the question type; the switch routes the question to the specialized model for that type, which directly outputs the final answer.

The second level can include differently designed models, where each model fits well for one or more question types, or the same model but fine-tuned with different hyper-parameters to be suitable for such a question type. The second level used the ELECTRA-SWIN and GS-ELECTRA-SWIN VQA models, as presented in Figure 3. The explanation of each transformer is given below.

**Figure 4:**
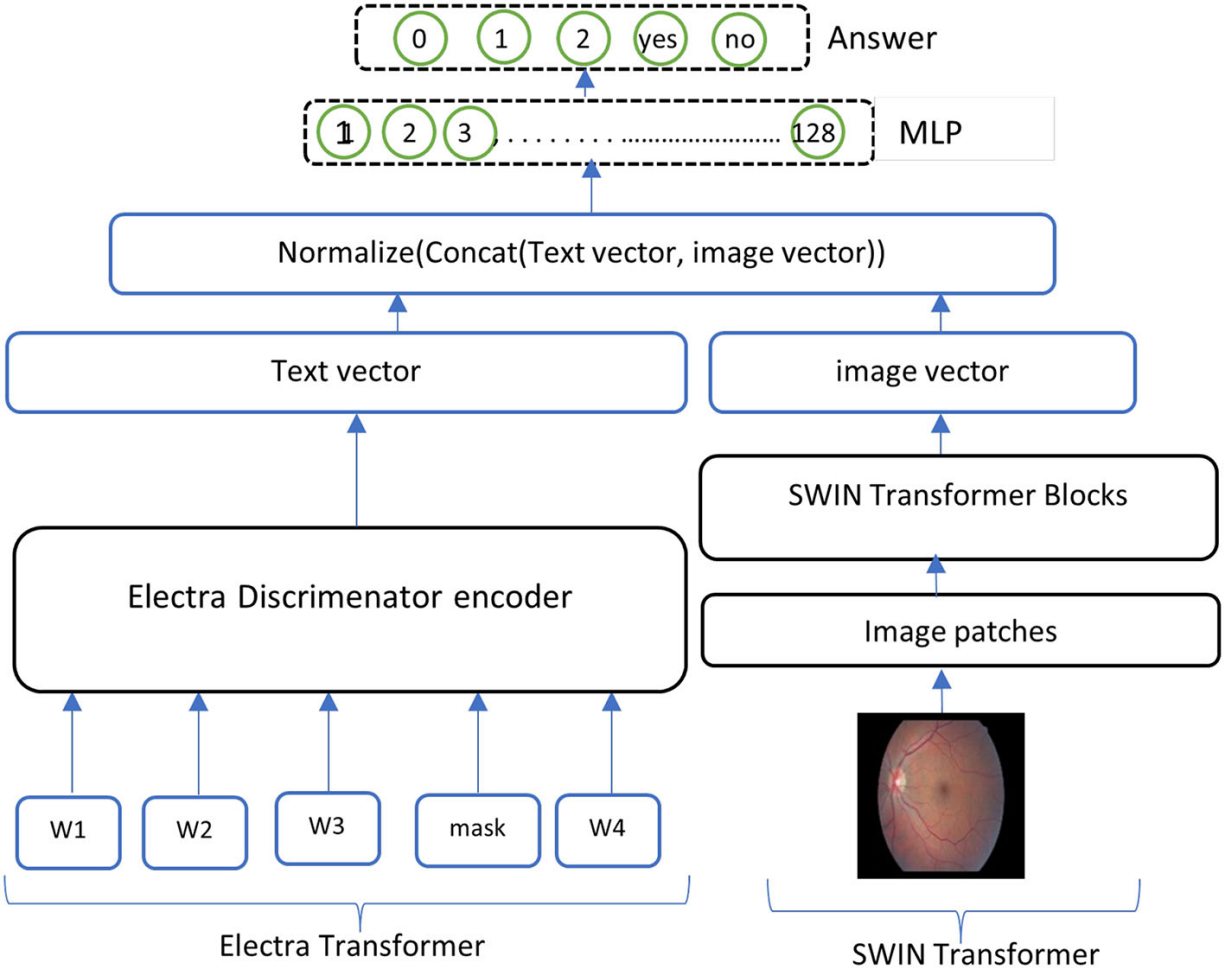
Overall structure of the ELECTRA-SWIN model. The input question is encoded using the ELECTRA discriminator, while the image is processed through the Swin Transformer. The resulting textual and visual feature representations are normalized, concatenated, and passed through an MLP to generate the final answer.

- **ELECTRA-SWIN** The proposed VQA model combines ELECTRA and Swin Transformers to extract text and visual features. The ELECTRA model is implemented to extract informative text features from the input question, while the Swin Transformer captures salient visual features from the corresponding image. The extracted text and visual features are subsequently combined and normalized to ensure they are on a similar scale. Finally, the normalized, concatenated features are passed to a MLP network, which classifies the answer based on the integrated information from both modalities. The general structure of the ELECTRA-SWIN model is shown in Figure 4. Algorithms 2 and 3 show the ELECTRA and SWIN features extraction respectively.
  The essential advantage of this architecture is its ability to leverage the robust feature extraction capabilities of ELECTRA and Swin Transformer, which have demonstrated SOTA performance on various NLP and computer-vision tasks. By fusing the text and visual features and passing them through an MLP, the model can effectively analyze the input image and the question to determine the accurate answer. This method offers an adaptable and scalable way to handle various possible answer choices, making it well-suited for diverse VQA scenarios.
- **GS-ELECTRA-SWIN** The ELECTRA-SWIN model is produced from the optimal selection criterion (Al-Hadhrami et al., 2023), which selects the model exhibiting the highest validation accuracy throughout the training. It can be mathematically written as follow in Equation (5):

(5) $$\begin{array}{l} f\left(x,{\text{argmax}}_{i}\text{ValAcc}\left(\theta_{i}\right)\right) \end{array}$$

The GS-ELECTRA-SWIN model integrates the greedy soup technique with the ELECTRA-SWIN model. The final model is chosen according to the models generated during the training phase, significantly impacting the average of all notable validation accuracies of fine-tuned models. The mathematical formulation of the model is as:
Let *M* = {*m*_1_, *m*_2_, …, *m*_*n*_} and θ = {θ_1_, θ_2_, …, θ_*n*_} represent the number of models and their corresponding parameters, accordingly. Additionally, consider θ−*SoupIngredients* = {θ_1_, θ_2_, …, θ_*k*_} and *M*_*k*_ = {*m*_*k*1_, *m*_*k*2_, …, *m*_*kk*_} as the set of selected parameters or the soup ingredients of the models under evaluation. At each validation computation step *i*, model *m*_*i*_ is included if its validation accuracy satisfies the condition *valAcc*(*m*_*i*_∪*m*_*kk*_)>min(*m*_*kk*_). The models in *M*_*k*_ are arranged in decreasing order based on their *valAcc* scores. Among the models in *M*_*k*_ and their corresponding θ−*SoupIngredients* parameters, a model is selected for fusion if, for each step from *i* to *k*, the validation accuracy *valAcc*(*sg*_*i*−1_∪{θ_*i*_}) exceeds *valAcc*(θ−*SoupIngredients*_*i*−1_). Let θ−*SoupIngredients*_*i*_ denote the set of *j* selected models. The final model parameters θ′ are computed as the average of the parameters of the chosen θ−*SoupIngredients* models, calculated as in Equation (6):

(6) $$\begin{array}{l} \theta^{\prime }=\frac{1}{j}\overset{j}{\underset{i=1}{\sum }}\theta_{i} \end{array}$$

**Figure 5:**
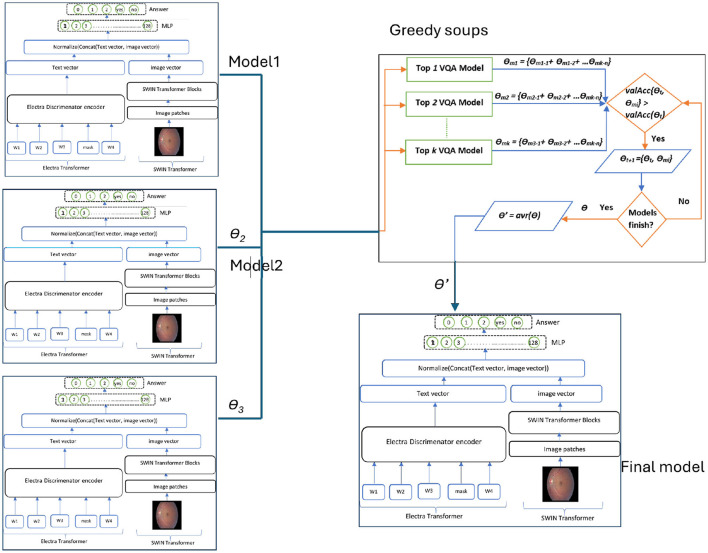
Overall structure of the GS-ELECTRA-SWIN model. Three independently trained ELECTRA-SWIN models, each with different training epochs, are combined using the greedy-soup technique to produce the final ensemble model.

The presented model depends on the top three leading models (*k* = 3), with validation evaluated at midway and at the end of each epoch. Algorithm 5 details the greedy soup algorithm for fusing three models with varying hyperparameters. Figure 5 illustrates the overarching greedy soup framework for merging three models.

- **Switch function** The switch function is tasked with determining whether to switch the input question and image to the appropriate model in the subsequent stage or to provide the final answer to the visual question. Equations 3, 4 outline the mathematical process involved in making this decision. Algorithm 4 shows the switch function procedure.

**Algorithm 2:** Feature extraction from ELECTRA-Base transformer.

**Require:** Input text *X*, pre-trained ELECTRA-Base model *M*
**Ensure:** Output feature vector *F*∈ℝ^*D*^
1: Tokenize input text and add special tokens for classification: *T* = [*CLS*]+*X*+[*SEP*]
2: Convert tokens to their corresponding token IDs: *I* = tokenizer(*T*)
3: Pass input token IDs through the pre-trained ELECTRA-Base model to derive the final hidden state: *H* = *M*(*I*)
4: Extract the final hidden-state of the special [CLS] token as the output feature vector: *F* = *H*_1, :_, where *H*_1, :_ denotes the first row of the hidden state matrix *H*
5: **Return** Output feature vector *F*

**Algorithm 3:** Feature extraction from SWIN-Base transformer.

**Require:** Input image *X*∈ℝ^*H*×*W*×*C*^, pre-trained SWIN-Base model *M*
**Ensure:** Output feature vector *F*∈ℝ^*D*^
1: Normalize input image: $X^{\prime }=\frac{X-\mu }{\sigma }$
2: Pad input image to a multiple of the patch size: *X*″ = ZeroPad(*X*′, *P*)
3: Split input image into non-overlapping patches of size *P*: $X_{i}=X^{\prime \prime }\left[p_{i}\right]$, where *p*_*i*_ denotes the coordinates of the *i*-th patch
4: Embed each patch using a learnable embedding layer: *E*_*i*_ = *W*_*emb*_(*X*_*i*_), where *W*_*emb*_ is a learnable weight matrix
5: Add positional embeddings to each patch embedding: *E*_*i*_ = *E*_*i*_+*P*_*i*_, where *P*_*i*_ is a learnable positional embedding
6: Pass input patches through the pre-trained SWIN-Base model to obtain the final hidden state: *H* = *M*(*E*)
7: Apply a global average pooling function to the hidden state to obtain the output feature vector: $F=\frac{1}{N}\overset{N}{\underset{i=1}{\sum }}H_{i}$, where *N* is the total number of patches
8: **Return** Output feature vector *F*

**Algorithm 4:** Bi-level VQA with switch function.

**Require:** Image *I*, Question *Q*
**Ensure:** Final Answer *A*
1: *a*_1_←GS-ELECTRA-SWIN(*I, Q*) {Level 1 prediction}
2: *decision*←SwitchFunction(*a*_1_) {Determine next action}
3: **if** *decision* = = final **then**
4: *A*←*a*_1_
5: **return** *A*
6: **else if** *decision* = = route_to_model1 **then**
7: *A*←ELECTRA-SWIN(*I, Q*) {Level 2 - Model 1}
8: **else if** *decision* = = route_to_model2 **then**
9: *A*←GS-ELECTRA-SWIN(*I, Q*) {Level 2 - Model 2}
10: **else**
11: *A*←GS-ELECTRA-SWIN(*I, Q*) {Level 2 - Model 3. Model 3 has different hyperparameters from Model.}
12: **end if**
13: **return** *A*

### 3.2 Training using greedy soup technique

The proposed multi-level VQA model employs pre-trained models for both textual and visual feature extraction. These models are fine-tuned using the designed Med-VQA dataset to adapt to the specific requirements of medical question-answering. To improve the generalization and performance of the model, the greedy soup method is applied for fine-tuning parameters. This technique integrates several fine-tuned models by fusing their parameters, thus providing a general and efficient configuration.

**Figure 6:**
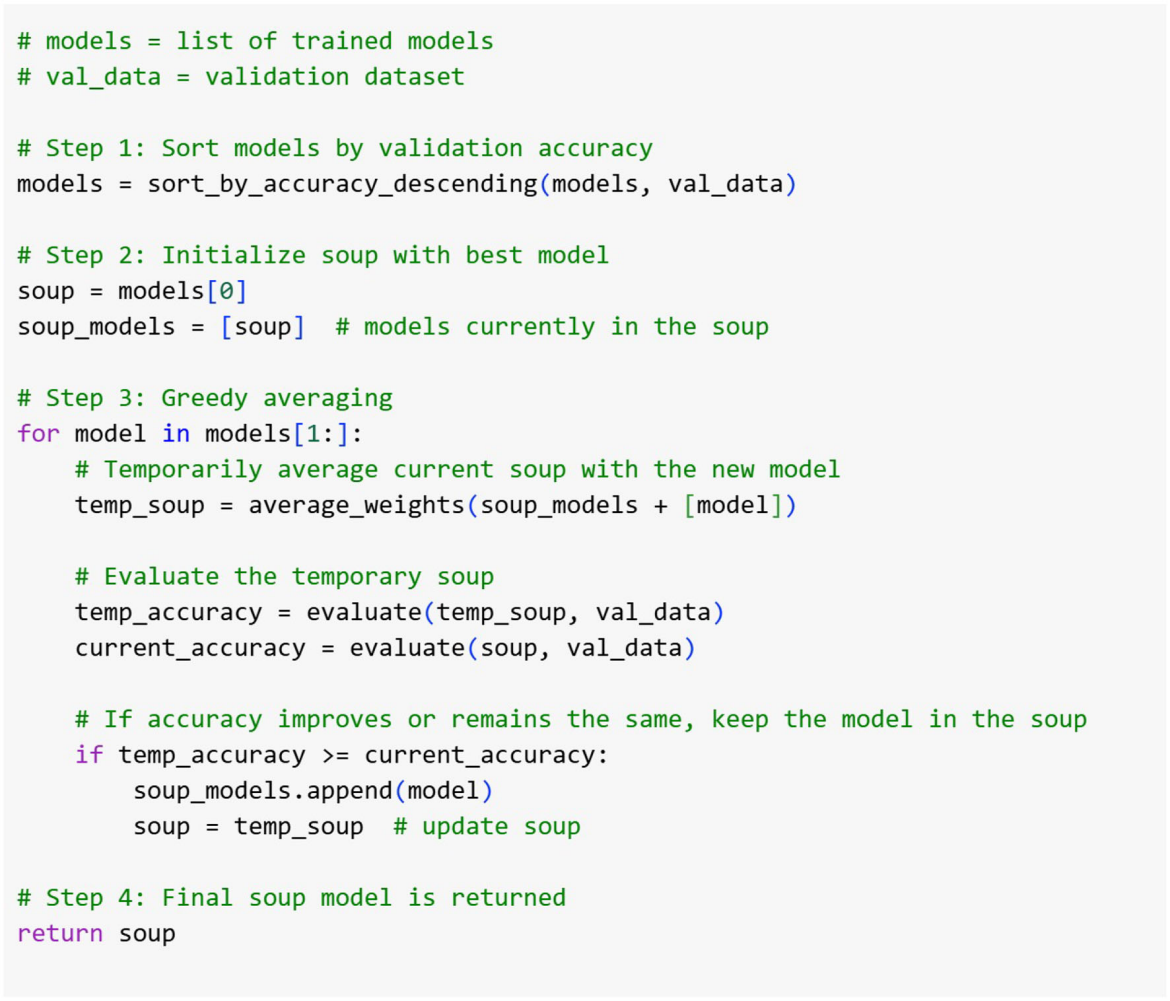
The Pseudo code of the greedy-soup ensemble technique, where the final model weights are generated based on the three most significant model weights regarding the validation accuracy.

During the training process, the model undergoes multiple rounds of fine-tuning, and validation accuracy is calculated at each stage. The final parameters are derived by averaging the weights of the best *k* models, selected according to their validation performance. This fusion technique significantly improves the generalization capability of the model, minimizing overfitting and enhancing performance. For this research, the final model is generated using the top three fine-tuned configurations, with different hyperparameters, integrated through the greedy soup technique. This process is illustrated in the pseudocode (Figure 6), Algorithm 5 and depicted in the flowchart in Figure 5.

**Algorithm 5:** Greedy soup for model ensembling

**Require:** $M=\left\{\theta_{1},\theta_{2},\ldots ,\theta_{N}\right\}$: List of model checkpoints, $D_{\text{val}}$: Validation dataset, $\text{Acc}\left(\theta ,D_{\text{val}}\right)$: Accuracy function
**Ensure:** θ_soup_: Final averaged model
1: Sort M by descending accuracy:
2: $\text{Acc}\left(\theta_{1},D_{\text{val}}\right)\geq \text{Acc}\left(\theta_{2},D_{\text{val}}\right)\geq \cdots \geq \text{Acc}\left(\theta_{N},D_{\text{val}}\right)$
3: Initialize soup set: $S\leftarrow \left\{\theta_{1}\right\}$
4: Initialize soup model: θ_soup_←θ_1_
5: **for** *i* = 2 to *N* **do**
6: Compute temporary average: $\theta^{\prime }\leftarrow \frac{1}{|S|+1}\left(\underset{\theta \in S}{\sum }\theta +\theta_{i}\right)$
7: **if** $\text{Acc}\left(\theta^{\prime },D_{\text{val}}\right)\geq \text{Acc}\left(\theta_{\text{soup}},D_{\text{val}}\right)$ **then**
8: $S\leftarrow S\cup \left\{\theta_{i}\right\}$
9: $\theta_{\text{soup}}\leftarrow \theta^{\prime }$
10: **end if**
11: **end for**
12: **return** θ_soup_

Let Θ_1_, Θ_2_, and Θ_3_ denote the parameters of the visual, textual, and MLP components, respectively, while *k* denotes the number of models used for parameter fusion. The parameter $\Theta_{i}^{\left(j\right)}$ refers to the parameters of the *j*^th^ model in the *i*^th^ component. The combined parameter Θ for the final model is calculated as follows in Equation 7:


(7) $$\begin{array}{l} \Theta^{\left(1\right)}=\left\{\Theta_{1}^{\left(1\right)},\Theta_{2}^{\left(1\right)},\Theta_{3}^{\left(1\right)}\right\},\\ \Theta^{\left(2\right)}=\left\{\Theta_{1}^{\left(2\right)},\Theta_{2}^{\left(2\right)},\Theta_{3}^{\left(2\right)}\right\},\\ \Theta^{\left(3\right)}=\left\{\Theta_{1}^{\left(3\right)},\Theta_{2}^{\left(3\right)},\Theta_{3}^{\left(3\right)}\right\},\\ \;\vdots \\ \Theta^{\left(k\right)}=\left\{\Theta_{1}^{\left(k\right)},\Theta_{2}^{\left(k\right)},\Theta_{3}^{\left(k\right)}\right\} \end{array}$$

The final fused parameters *Theta* are computed using the following Equation 8:


(8) $$\begin{array}{l} \Theta =\frac{1}{k}\overset{k}{\underset{j=1}{\sum }}\overset{3}{\underset{i=1}{\sum }}\Theta_{i}^{\left(j\right)}. \end{array}$$

The combination of pre-trained backbones results in multiple concatenated configurations, which are normalized and processed through an MLP for final classification. These configurations are fused using the greedy soup technique to enhance performance and robustness, as demonstrated in Al-Hadhrami et al. (2023). Figure 5 illustrates the overall structure of the model integrated using the greedy soup method. Other configurations follow the same structure, replacing the pre-trained models used for feature extraction. This training strategy ensures that the final model effectively handles diverse question types, leveraging hierarchical VQA architecture and robust parameter fusion to deliver accurate and reliable predictions in Med-VQA tasks.

### 3.3 Accessibility considerations and system framework

This work proposes a multi-level Med-VQA framework aimed at enhancing accessibility for visually impaired users. The primary focus of the current study is on developing and validating the underlying machine learning models and architectural design, rather than delivering a fully integrated end-user system. The proposed framework provides a modular and extensible structure that demonstrates how various components–such as question classification, visual feature extraction, and answer generation—can be combined effectively. This modularity enables potential integration with accessible platforms in the future, including mobile and web applications that support assistive technologies like screen readers, Braille displays, and voice input/output systems. Accessibility considerations in the framework are informed by established standards, including the Web Content Accessibility Guidelines (WCAG) (W3C, 2023) and ISO 9241 (International Organization for Standardization, 2008), which provide internationally recognized guidelines for accessible system design. While the framework lays the technical groundwork, actual integration into real-world accessible interfaces and user-facing applications remains future work. Prior research underscores the importance of tailored interface solutions for visually impaired users. For example, Alhadhrami et al. (2015) showed that adaptive interfaces coupled with multimodal feedback significantly improve spatial awareness and usability. Similarly, recent studies highlight the benefits of embedding VQA capabilities into intelligent assistive devices such as wearable smart glasses and voice-controlled platforms to enhance user autonomy (Ainary, 2025).

To ensure practical accessibility impact, future efforts will include participatory evaluations with visually impaired individuals and clinical professionals. These studies will assess task efficiency, user satisfaction, and interaction quality through metrics such as time-to-answer, error rates, and voice/haptic response accuracy. Additionally, a prototype user interface featuring multimodal interaction (voice and haptics) is planned to explore usability and contextual adaptation further.

**Figure 7:**
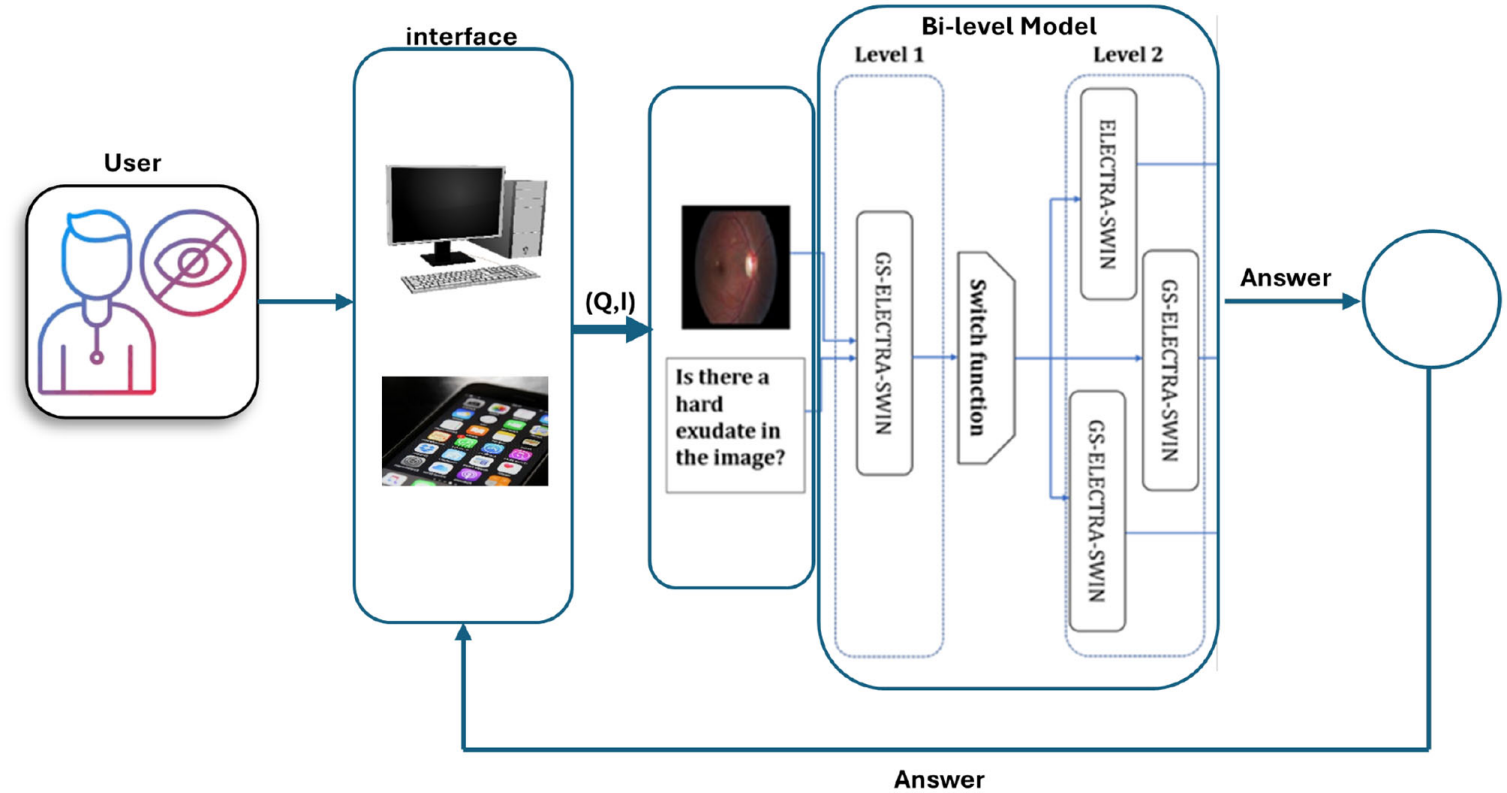
Framework architecture of the DR-VQR system. The user uploads a retinal image through the website or the iOS/Android application, formulates a related question, and submits the request. The query is processed by the bi-level VQA model, which generates an answer. The result is then displayed to the user via the mobile application or web interface.

In summary, the presented framework serves as a foundational architecture that outlines how Med-VQA components can be systematically integrated to support accessibility. The subsequent stages of research will focus on system integration, user-centered design, and rigorous validation to translate this framework into effective assistive technologies for visually impaired users. Figure 7 illustrates the DR VQR system framework architecture, which allows visually impaired users to create personal accounts to store their questions and answers. Additionally, the system can be accessed both online and offline.

## 4 Evaluation protocol

### 4.1 Experimental environment configuration

The models undergo training on a premium Google Colab utilizing NVIDIA A100-SXM4-40 GB (Nvidia Corporation, Santa Clara, CA, USA) with 80 GB RAM or an NVIDIA Tesla T4 with15GB and 25 GB or 51 GB RAM. The optimization function utilizes AdamW with a learning rate of 1.0 × 10^−3^ and weight decay of 0.9. A fixed random seed (seed = 42) was configured to ensure deterministic behavior and reproducibility of the results. Consequently, the outputs remain consistent across runs, resulting in zero variance in the reported scores. While traditional statistical significance tests rely on variability across multiple runs, in our setup, reproducibility implies that even a small performance gain (e.g., 0.1%) is meaningful and reliable under the same evaluation conditions. More details about model configuration are listed in Table A1 in the Appendix.

### 4.2 Assessment criteria

Model performance is evaluated based on the calculation of metrics: precision, model accuracy, F1 score, recall (Powers, 2011), macro-average recall, macro-average precision, weighted average precision, macro-average F1 score, weighted average F1 score, and weighted average recall (learn developers, 2024). The performance metrics utilized to evaluate the model and compare the findings with other state-of-the-art models are presented below. The equation representing each metric is given below.

- **Accuracy:** This is determined using the formula shown below Equation 9:

(9) $$\begin{array}{l} \text{Accuracy}=\frac{\text{TN + TP}}{\text{TN + TP + FN + FP}} \end{array}$$

True positives (TP) refer to actual positive instances that are correctly predicted by the model as positive. True Negatives (TN) represent the negative instances accurately classified as negative. False positives (FP) occur when negative instances are incorrectly predicted as positive. Lastly, false negatives (FN) denote positive instances that the model mistakenly classifies as negative.

- **Precision:** measures the ratio of correctly predicted true positive instances relative to the total predicted positive instances. This metric is defined as in Equation 10:

(10) $$\begin{array}{l} \text{Precision}=\frac{TP}{FP+TP} \end{array}$$

- **Recall sensitivity:** quantifies the proportion of correctly predicted positive instances relative to the total actual positive instances. This metric is measured by Equation 11:

(11) $$\begin{array}{l} \text{Recall}=\frac{TP}{TP+FN} \end{array}$$

- **F1-score:** The F1-score assesses a model's accuracy in detecting positive instances by determining the harmonic mean of precision and recall. It is computed as in Equation 12:

(12) $$\begin{array}{l} F1=2\times \frac{recall\times precision}{recall+precision} \end{array}$$

- **Average macro accuracy:** The macro average accuracy assesses the model's performance by calculating the accuracy of each class independently and after that averaging these accuracies. The macro accuracy average formula is given in: (13):

(13) $$\begin{array}{l} \text{Macro Accuracy Average}=\frac{1}{C}\overset{C}{\underset{c=1}{\sum }}\frac{TP_{c}}{TP_{c}+FP_{c}} \end{array}$$

where *C* denotes the total number of classes, *TP*_*c*_ is the number of true positives for class *c*, and *FP*_*c*_ is the number of false positives for class *c* .

- **Weighted average accuracy:** calculates the average accuracy for individual classes, considering the class frequencies in the dataset to assign weights. The weighted average accuracy equation is given by Equation 14:

(14) $$\begin{array}{l} \text{Weighted average accuracy}=\frac{n_{c}\times {\text{TP}}_{c}}{\text{n}} \end{array}$$

Where *n* is the total number of samples in the dataset and *n*_*c*_ is the number of samples belonging to class *c*. The *TP*_*c*_ are as defined above.

### 4.3 Dataset

In this study, the Diabetic Macular Edema (DME) (Tascon-Morales et al., 2022) is used, which was automatically generated from the Indian Diabetic Retinopathy Image Dataset (IDRiD) (Porwal et al., 2018) and the e-Ophta dataset (Decenciere et al., 2013). It comprises 13,470 question-answer pairs and 679 images, divided into 433 images and 9,779 question-answer pairs for training, 134 images and 2380 pairs for validation, and 112 images and 1,311 pairs for testing.

The dataset includes questions regarding hard exudates, optic discs, and the grading of exudates. The dataset includes a question asking whether a hard exudate is present in the image or a specific region of the image. If a hard exudate is present, the answer is labeled as “Yes”; otherwise, it is labeled as “No”. The grading system classifies hard exudates on the retina as follows: grade 0 indicates no presence of hard exudates, grade 1 signifies hard exudates located in the peripheral retina, and grade 2 denotes the presence of hard macular exudates. Additionally, the dataset provides original images along with masks that highlight specific regions of the images, which must be utilized for pre-training. Table 1 presents the distribution of classes in the training, validation, and testing datasets.

**Table 1:** Number of instances per answer for each part of the DME dataset.

**Set**	**Yes**	**No**	**0**	**1**	**2**	**Total**
Train	4,713	4,639	166	41	220	9,779
Val	1,151	1,123	39	8	59	2,380
Test	530	650	49	15	67	1,311
Total	6,394	6,412	254	64	346	13,470

The DME dataset consists of four distinct types of questions, each with different levels of complexity:

**Whole:** e.g., “Are there hard exudates in this image?”—requires a binary decision at the image level.

**Region:** e.g., “Are there hard exudates in the region?”—focuses on a predefined mask in the image and typically requires less complex reasoning since the region is already localized.

**Fovea:** e.g., “Are there hard exudates in the fovea?”—requires detection of exudates and precise spatial reasoning to determine whether they fall within the foveal region.

**Grade:** e.g., “What is the diabetic macular edema grade for this image?”—involves multi-class classification based on exudate presence and location.

While Region-type questions are the most frequent, Fovea and Grade questions are the most complex. They first require the system to detect the presence of hard exudates and then localize them accurately relative to the foveal region. This question complexity demands multi-step spatial understanding and is more aligned with clinical decision-making processes. The differences in question complexity provide a valuable framework for evaluating the robustness and reasoning capabilities of VQA models. The distribution of question types across each part of the dataset is shown in Table 2.

**Table 2:** Number of instances per question type for each part of the DME dataset.

**Question type**	**Train**	**Validation**	**Test**	**Total**
Grade	427	106	131	664
Macula (Fovea)	427	106	131	664
Whole	427	106	131	664
Region	8,498	2,062	918	11,478
Total	9,779	2,380	1,311	13,470

**Figure 8:**
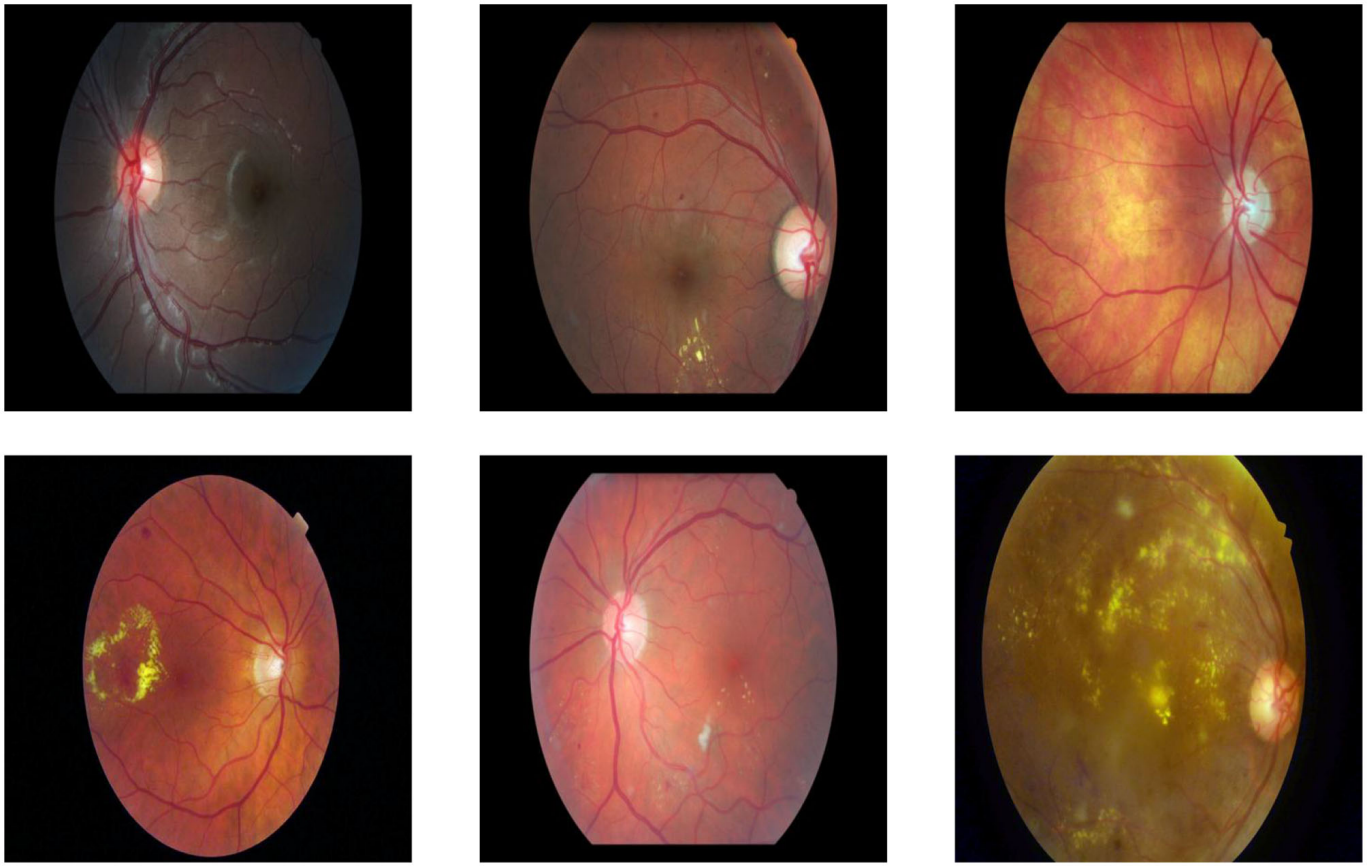
Examples of dataset images captured under varying conditions, including differences in illumination, clarity, noise levels, size, and object positioning.

The dataset includes retinal images captured under varied conditions, such as differences in illumination, patient eye positions, and inherent noise. This variability reflects realistic clinical scenarios and adds robustness to the evaluation of the proposed VQA framework. Figure 8 shows samples on dataset images.

### 4.4 Test significance and impact of seed setting

Randomness in machine learning experiments, such as weight initialization and data shuffling, can lead to variability in model performance. We used a fixed random seed during training and evaluation to mitigate this. Setting a random seed enhances the reproducibility of experiments and ensures that the reported results are stable and not artifacts of random initialization.

To evaluate the impact of the random seed on initial weight settings, we conducted five independent experiments using the base model. All experiments shared the same architecture and training configuration, differing only in the random seed used for weight initialization. The selected seeds were chosen randomly: 10, 23, 42, 70, and 100. Table 3 reports the Accuracy obtained for each seed. The accuracies ranged from 85.89% to 87.41%, with a mean accuracy of **86.32%** and a standard deviation of **0.62**. We compared these results against the state-of-the-art (SOTA) results reported by (Tascon-Morales et al., 2022, 2023), which achieved 83.00% and 83.69% accuracy, respectively.

**Table 3:** Accuracy results for different random seeds compared to the SOTA baseline of Tascon-Morales et al. (2022, 2023).

**Seed**	**Accuracy (%)**
10	86.04
23	86.12
42	**87.41**
70	86.12
100	85.89
**Mean**	86.32 ± 0.62
***p*** **-value**	0.0007
Tascon-Morales et al. (2022)	83.00
Tascon-Morales et al. (2023)	83.59 ± 0.69

Among the tested seeds, seed 42 achieved the highest Accuracy (87.41%), and we adopted this setting for all subsequent experiments, including the first-level classifier and the other components in our framework.

To determine whether the improvements over Tascon-Morales et al. (2023) are statistically significant, we performed a paired two-tailed t-test using the accuracies of our five seed experiments and the baseline of 83.69% (Tascon-Morales et al., 2023). The t-test yielded a t-statistic of 9.49 and a *p*-value of 0.0007, indicating that the performance improvement is statistically significant at the 0.01 level.

These results justify our choice of seed 42 for all subsequent experiments, as it consistently provided the best initialization and final Accuracy. Furthermore, the statistical test confirms that our method achieves a significant improvement over previous works.

### 4.5 Result and analysis

Our proposed model employs a two-level system. The initial level comprises a VQA model that inputs an image and a question as input and outputs the question type. We fine-tuned the model using the DME dataset, which contains four question types: grade, whole, region, and fovea or macula.

During this stage, we fine-tuned the GS-SWIN-ELECTRA model with a batch size of 32 and learning rate of 1.0 × 10^−4^. Instead of answers, we replaced the classes with the question types. Remarkably, the model quickly converged within the first epoch, allowing us to train it just once. The model exhibited remarkable performance, achieving 99.85% for all performance metrics.

**Figure 9:**
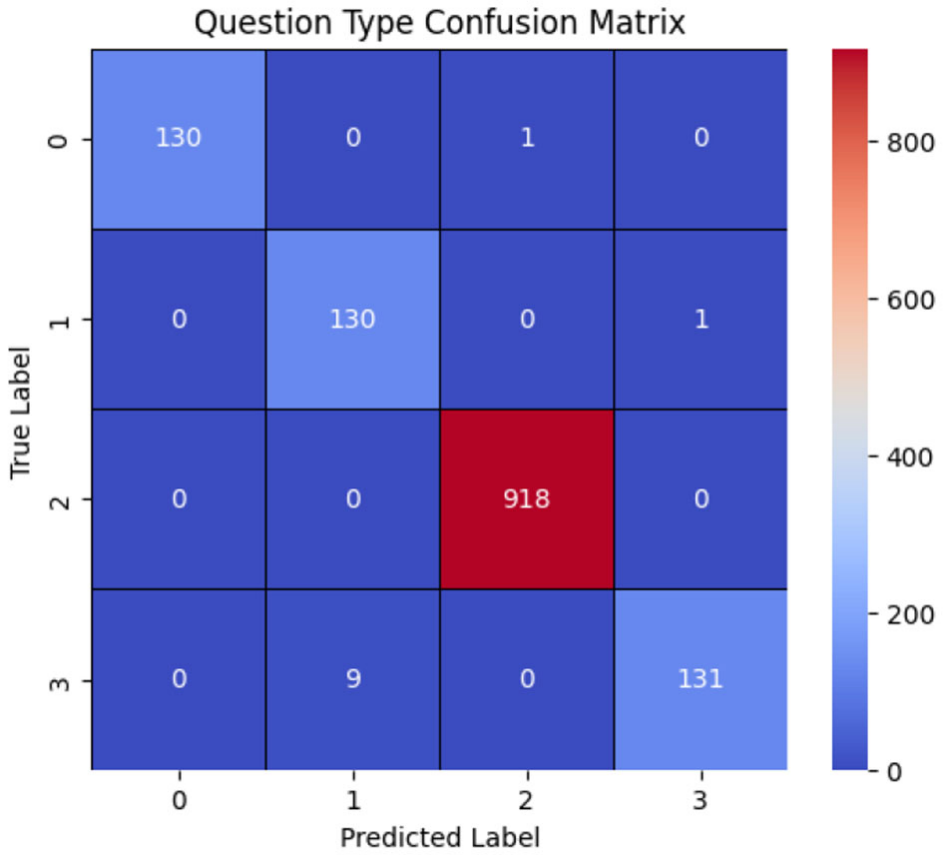
Onfusion matrix of the question-type classification model, where labels 0, 1, 2, and 3 correspond to whole, grade, fovea, and region, respectively. The results show that the model correctly classifies most question types, with the primary misclassification occurring when region questions are predicted as grade.

These results arise from several characteristics of the dataset. Firstly, the number of questions is relatively limited. Moreover, question types such as grade and fovea are directly reflected in the question text itself. In contrast, questions related to regions and wholes do not have distinct textual characteristics for classification. Instead, the classification between these two question types relies on the image provided, distinguishing between a whole image and a specific region based on the applied mask. Table 4 presents the model's performance, while Figure 9 illustrates the model's confusion matrix.

**Table 4:** The result of the first-level model.

**Answer**	**Precision**	**Recall**	**F1-Score**	**Instances no**.
Fovea	1.0000	0.9924	0.9962	131
Grade	1.0000	0.9924	0.9962	131
Region	0.9989	1.0000	0.9995	918
Whole	0.9924	1.0000	0.9962	131
**Accuracy**	–	–	**0.9985**	**1,311**
**Macro Avg**	0.9978	0.9962	0.9970	1,311
**Weighted Avg**	0.9985	0.9985	0.9985	1,311

Achieving high performance in the lower levels is critical in our proposed multi-level framework, as these levels route visual questions to the appropriate upper levels. In our case, the first-level achieved 99.85% accuracy, which we attribute to the abovementioned reasons. However, this may not generalize to all problem domains. However, this high accuracy may not generalize across different problem domains. This sensitivity to the first-level performance highlights a potential limitation in our approach: the overall system's effectiveness depends on the performance of the initial routing decisions.

We also recognize a limitation in our statistical methods because we used a fixed random seed for all experiments. This approach guarantees reproducibility, but it removes natural variation and hinders the accurate estimation of variance or significance. Therefore, the reported improvements, like the 1% gain over SOTA baselines, should be viewed with caution. In future work, we intend to include repeated runs with different seeds and report confidence intervals to better evaluate performance stability and significance.

Moreover, our evaluation focused specifically on diabetic retinopathy in the context of disability, using the only publicly available dataset in this domain. We did not validate the framework on other datasets. In future work, we will expand the dataset to include more diverse DR cases and explore cross-lingual generalization by applying the method to data in additional languages.

Our current evaluation is limited to the DME-VQA dataset, which may constrain the generalizability of our findings. While this dataset is the only publicly available benchmark for diabetic retinopathy visual question answering that focuses on accessibility, future work will tackle this issue by doing cross-dataset evaluations with resources like extending the DME-VQA dataset or new public ones. Additionally, to measure real-world impact, we plan to include usability testing with visually impaired users.

**Figure 10:**
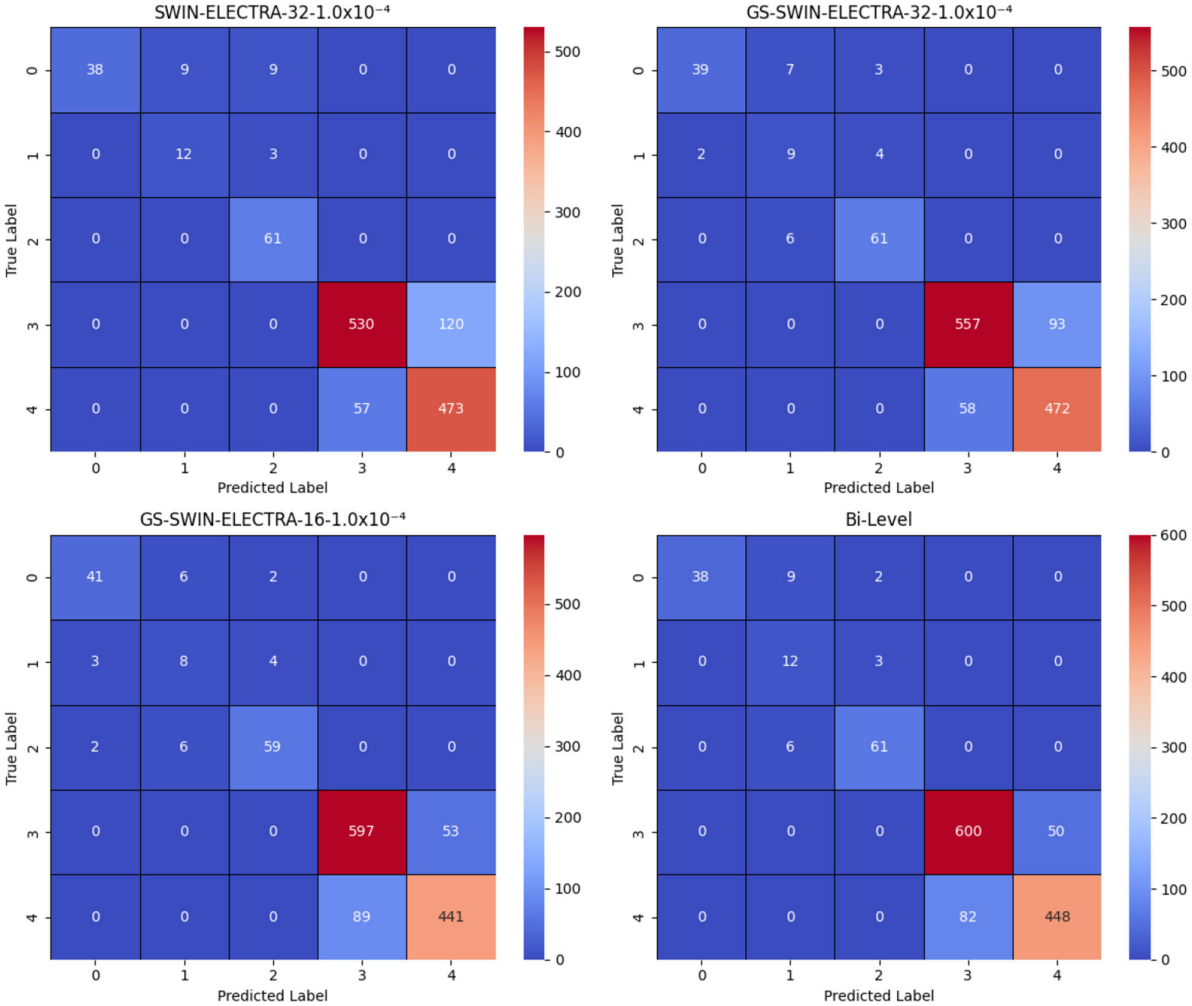
Confusion matrices for the SWIN-ELECTRA and GS-SWIN-ELECTRA models, where 1.0 × 10^−4^ denotes the learning rate, and 32 and 16 denote the batch sizes. In the answer labels, “3” corresponds to No and “4” corresponds to Yes. The results clearly show that the bi-level model performs equal to or better than its strongest component across most answers, with the exception of answer “0”, where the GS-SWIN-ELECTRA model achieves superior performance, correctly identifying 41 cases.

After generating a prediction at the first-level, the model passes it through a switch function, which routes the visual question to the appropriate model at the second level. The second level comprises three models: SWIN-ELECTRA (with a batch size of 32 and a learning rate of 1.0 × 10^−4^), GS-SWIN-ELECTRA (with a batch size of 32 and a learning rate of 1.0 × 10^−4^), and GS-SWIN-ELECTRA (with a batch size of 16 and a learning rate of 1.0 × 10^−4^). These models are specifically designed to handle different question types: grade, whole, fovea, and region, respectively. Table 5 provides insights into the performance of the bi-level model across various evaluation metrics. To further visualize the prediction outcomes, Figure 10 displays the confusion matrix, illustrating the predictions for each answer.

**Table 5:** The result of the bi-level model.

**Answer**	**Precision**	**Recall**	**F1-Score**	**Instances no**.
0	1.0000	0.7755	0.8736	49
1	0.4444	0.8000	0.5714	15
2	0.9242	0.9104	0.9173	67
No	0.8798	0.9231	0.9009	650
Yes	0.8996	0.8453	0.8716	530
**Accuracy**	–	–	**0.8841**	**1,311**
**Macro Avg**	0.8296	0.8509	0.8270	1,311
**Weighted Avg**	0.8896	0.8841	0.8851	1,311

To evaluate the effectiveness of our model, we analyze its performance to that of its individual components. This evaluation was conducted using several performance metrics, including F1 score, recall, and precision for each answer, as well as model accuracy, macro average precision, macro average recall, macro average F1 score, weighted average precision, weighted average recall, and weighted average F1-score. Tables 6, 7 present a comprehensive comparison of the proposed model and its individual components across these evaluation metrics. Furthermore, Figure 10 illustrates the confusion matrices, highlighting how each model distributed its predicted answers.

**Table 6:** The result comparison per each answer for Bi-level model and its model components, where Model-1 is the SWIN-ELECTRA model with a 32 batch size and 1.0 × 10^−4^ learning rate, Model-2 is the GS-SWIN-ELECTRA model with 32 batch size and 1.0 × 10^−4^ learning rate, Model-3 is the GS-SWIN-ELECTRA model with 16 batch size and 1.0 × 10^−4^ learning rate, and Model-4 is the Bi-level model with 32 batch size and 1.0 × 10^−4^ learning rate

**Metric**	**Answer**	**Model 1**	**Model 2**	**Model 3**	**Model 4**	**Samples#**
Precision	0	**1.000**	0.9512	0.8913	**1.0000**	49
	1	**0.4444**	0.4091	0.4000	**0.4444**	15
	2	**0.9242**	0.8971	0.9077	**0.9242**	67
	no	0.9029	**0.9057**	0.8703	0.8798	650
	yes	0.7976	0.8354	0.8927	**0.8996**	530
Recall	0	0.7755	0.7959	**0.8367**	0.7755	49
	1	**0.8000**	0.6000	0.5333	**0.8000**	15
	2	**0.9104**	**0.9104**	0.8806	**0.9104**	67
	no	0.8154	0.8569	0.9185	0.9231	650
	yes	**0.8925**	0.8906	0.8321	0.8453	530
F1-score	0	**0.8736**	0.8667	0.8632	**0.8736**	49
	1	**0.5714**	0.4865	0.4571	**0.5714**	15
	2	**0.9173**	0.9037	0.8939	**0.9173**	67
	no	0.8569	0.8806	0.8937	**0.9009**	650
	yes	0.8424	0.8621	0.8613	**0.8716**	530

**Table 7:** The result comparison of Bi-level model and its model components.

**Metric**	**Model 1 Al-Hadhrami et al. (2023)**	**Model 2 Al-Hadhrami et al. (2023)**	**Model 3 Al-Hadhrami et al. (2023)**	**Model 4**
Macro avg Precision	0.8138	0.7997	0.7924	0.8296 ± 0.0189 *p*-value = 0.0017
Macro avg Recall	0.8388	0.8108	0.8002	0.8509 ± 0.0251 *p*-value = 0.0014
Macro avg F1-score	0.8123	0.7999	0.7939	0.8270 ± 0.0234 *p*-value = 0.0033
Weighted avg Precision	0.8598	0.8729	0.8767	0.8896 ± 0.0078 *p*-value = 0.0015
Weighted avg Recall	0.8497	0.8680	0.8741	0.8841 ± 0.0059 *p*-value = 0.0015
Weighted avg F1-score	0.8515	0.8693	0.8745	0.8851 ± 0.0067 *p*-value = 0.0017
Accuracy	0.8497	0.8680	0.8741	0.8841 ± 0.0059 *p*-value = 0.0015

Model 1 is the SWIN-ELECTRA model with 1.0 × 10^−4^ learning rate and 32 batch size (Al-Hadhrami et al., 2023). Model 2 is GS-SWIN-ELECTRA model with 1.0 × 10^−4^ learning rate and 32 batch size (Al-Hadhrami et al., 2023). Model 3 is the GS-SWIN-ELECTRA model with 1.0 × 10^−4^ learning rate and 16 batch size (Al-Hadhrami et al., 2023). Model 4 is the Bi-level model with 1.0 × 10^−4^ learning rate and 32 batch size.

The bi-level model consistently achieves higher accuracy for each question type compared to its component models. We selected the component models based on their superior performance in those specific question types. This strategy allowed the bilevel model to achieve the highest performance among the component models and improve its overall accuracy. For each question type, our proposed bi-level VQA model consistently achieves the highest accuracy compared to its individual component models, demonstrating its effectiveness and contributing to the best overall performance across the dataset. In Table 8, we present the performance comparison between the bi-level model and its component models, providing an insightful overview of their respective performances.

**Table 8:** The result comparison of Bi-level model and its model components based on question types, where Model-1 is the SWIN-ELECTRA model with 1.0 × 10^−4^ learning rate and 32 batch size, Model 2 is GS-SWIN-ELECTRA model with 1.0 × 10^−4^ learning rate and 32 batch size, Model-3 is the GS-SWIN-ELECTRA model with 1.0 × 10^−4^ learning rate and 16 batch size, Model 4 is the Bi-level model with 1.0 × 10^−4^ learning rate and 32 batch size.

**Model**	**Overall**	**Grade**	**Whole**	**Macula**	**Region**
SOTA 2022 Tascon-Morales et al. (2022)	83.49	80.69	84.96	87.18	83.16
SOTA 2023 Tascon-Morales et al. (2023)	83.59 ± 0.69	80.15 ± 0.95	86.22 ± 1.67	88.18 ± 1.07	82.62 ± 1.02
Model 1	84.97	**84.73**	90.84	85.29	83.22
Model 2	86.80	83.21	**92.37**	**90.84**	85.95
Model 3	87.41	82.44	88.55	87.02	**88.02**
Model 4 (proposed bi-level)	**88.41±** **0.0059**	**84.73** **±** **0.0125**	**92.37** **±** **0.0185**	**90.84** **±** **0.0211**	**88.02** **±** **0.0133**
	***p*** **-value= 0.0015**	***p*****-value:** **1.66 × 10^−8^**	***p*****-value:** **5.11 × 10^−8^**	***p*****-value:** **7.80 × 10^−8^**	***p*****-value:** **1.39 × 10^−8^**

In addition, the model is compared with SOTA 2022 (Tascon-Morales et al., 2022) and SOTA 2023 (Tascon-Morales et al., 2023).

Our framework we introduced in this work is designed to be modular and adaptable, enabling its application beyond the DME-VQA dataset.

Its generalizability stems from its core design, which emphasizes structured understanding and decomposition of the problem domain. The framework can be adapted to various medical imaging tasks or other vision-language problems by analyzing the dataset and identifying distinct question types or visual characteristics. The bi-level architecture offers flexible integration of specialized models for distinct sub-tasks, enabling its extension to new datasets with varying class or diagnostic objectives distributions. Furthermore, this decomposition strategy improves interpretability and reduces the learning complexity, especially in scenarios with limited annotated data. The framework enables more efficient learning by transforming a complex VQA task into smaller, more focused subtasks, potentially improving performance and generalization even when data is scarce.

Furthermore, we evaluated the model on a dataset that incorporates real-world variability, including noise, inconsistent illumination, and diverse imaging angles. This diversity contributes to the robustness and reliability of the proposed system in clinically realistic settings.

**Figure 11:**
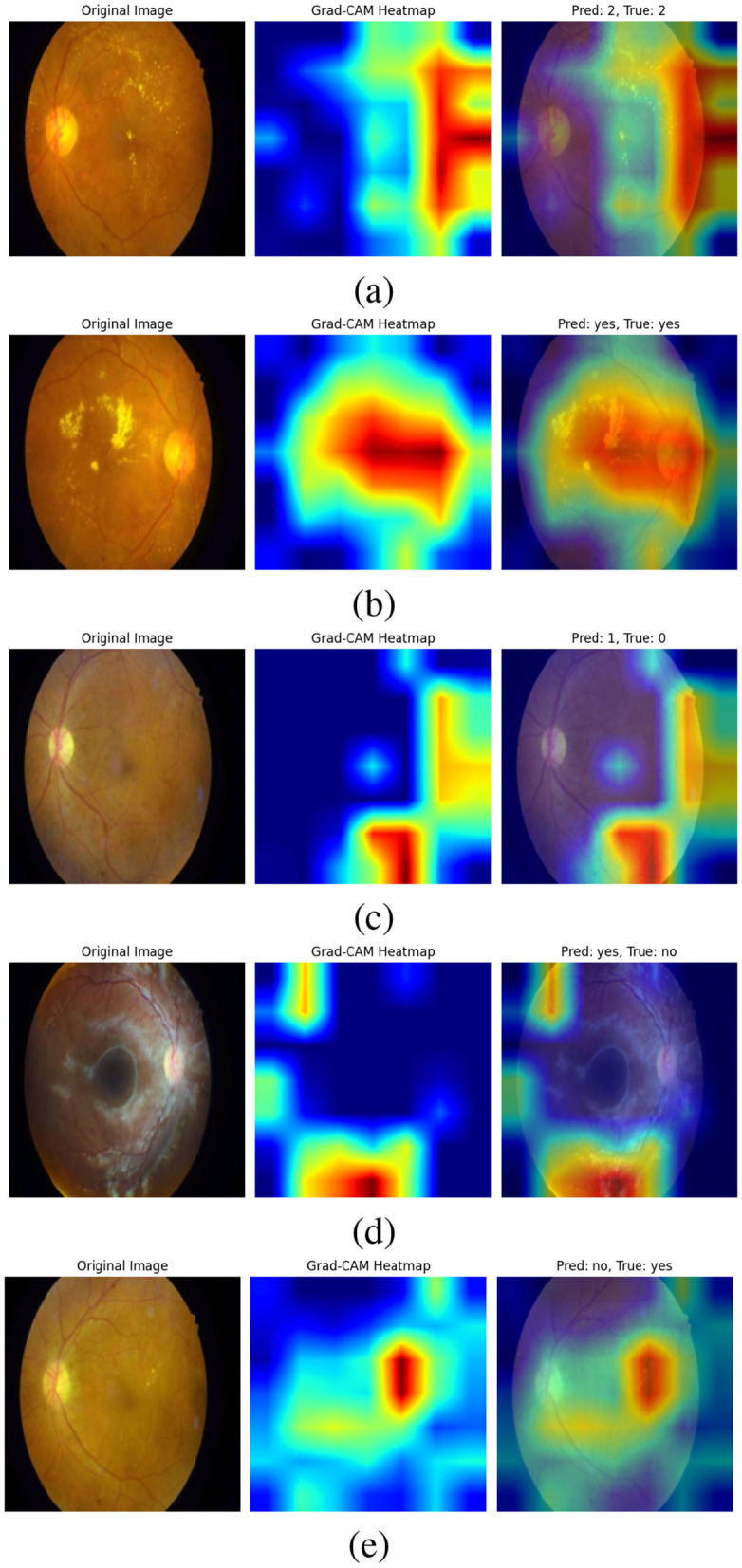
Grad-CAM visualizations of the bi-level VQA model (accuracy: 88.41%), highlighting its ability to attend to critical retinal regions for diabetic retinopathy classification. In correctly predicted cases **(a, b)**, the model focuses on key features such as hard exudates, aligning with ground truth labels. In misclassified cases **(c–e)**, attention is diverted to irrelevant regions or image noise, occasionally leading to errors. Notably, in **(e)**, the model attends correctly to the lesion but interprets it as “No”. These visualizations demonstrate both the robustness of the model and areas needing refinement to improve attention consistency.

Figure 11 Grad-CAM shows visualizations of our bi-level VQA model, which has an impressive accuracy of 88.41%, effectively highlight the model's ability to focus on critical regions in retinal images for diabetic retinopathy classification. In the correctly predicted cases, as shown in Figures 11a, b, the heatmaps show strong attention to essential features such as hard axudates, aligning with the ground truth labels. This finding demonstrates the model's capacity to identify and leverage key visual cues, further validating its robustness in making accurate predictions. These visualizations confirm that the model is consistently able to attend to relevant areas of the image, supporting its high performance.

On the other hand, the incorrect predictions appear to stem from the model focusing on irrelevant regions as shown in Figure 11c. In the case where the model predicts “yes” incorrectly, the heatmap shows attention to parts of the image that are not relevant to the key features of diabetic retinopathy, suggesting that the model might be misinterpreting image details. In the last image (d), the error could be attributed to image noise, which may have caused the model to focus on non-essential features, leading to an incorrect classification. In (e), the attention is correctly focused on the hard exudates, but it is interpreted as “no”. This case requires further analysis and study, which we will address in future work. These observations highlight areas for future work to refine the model's attention mechanism, improving its ability to focus on the most relevant features and reducing the impact of noise.

### 4.6 Ethical considerations for medical VQA

Ethical concerns in medical VQA include user privacy, deployment implications, and potential biases. Privacy safeguards are critical, as these systems handle sensitive patient data, requiring compliance with frameworks like HIPAA and informed consent protocols to protect autonomy and dignity (Majumder and Guerrini, 2016; Adeniyi et al., 2024; De Lusignan et al., 2015). Deployment strategies must prioritize equitable access, addressing cost barriers to ensure the widespread availability of these technologies (Adeniyi et al., 2024).

Algorithmic bias is another significant challenge, as it can lead to inequitable outcomes across diverse populations. Biases often arise from non-representative datasets or flawed model development processes, exacerbating healthcare disparities. Mitigation strategies include using diverse datasets, statistical debiasing methods, and rigorous validation through clinical trials (Smith et al., 2023; Cross et al., 2024). Addressing these ethical considerations ensures medical VQA systems contribute positively to society while minimizing risks.

## 5 Conclusion

Visual disabilities affect the ability of individuals to perceive and interpret visual information, highlighting the need for advanced solutions to solve these challenges. This paper introduces a multi-level VQA technique that leverages multiple VQA models for enhancing the VQA performance. We propose a bi-level, designed to enhance VQA performance. The bi-level model consists of two levels. The type of question is classified in the first-level, and the visual question is answered in the second level. The model employs a switch function to forward the visual question to the proper component model according to its question type. Through this multi-level VQA model, we demonstrate the efficacy of incorporating different levels and component models to enhance the accuracy of VQA systems. We believe this approach represents a step forward in making visual information more accessible to individuals with visual impairments.

Looking ahead, future work will focus on optimizing the number and structure of the levels to maximize performance. Exploring additional hierarchical levels may improve accuracy by enabling more fine-grained routing of visual questions. Moreover, we aim to conduct usability and accessibility evaluations involving users with visual impairments to validate the system's practical impact. Lastly, we plan to extend the framework to support multilingual datasets and evaluate its generalizability across diverse linguistic and demographic populations as new diabetic retinopathy VQA datasets become available. In addition, we aim to implement the full system and measure the user satisfication and system usibility.

## Data Availability

Publicly available datasets were analyzed in this study. This data can be found here: ZENODO repository at https://zenodo.org/records/6784358.

## Appendix

**Table A1:** Model configuration summary.

**Component**	**Configuration details**
**Model names**	fusion_mlp, hf_text, timm_image
**hf_text**	Checkpoint: local://hf_text Pooling Mode: cls Max Text Length: 512 Tokenizer: hf_auto Segment Num: 2 Insert SEP: true Text Aug Detect Length: 10
**timm_image**	Checkpoint: swin_base_patch4_window7_224 Mix Choice: all_logits Transforms: resize_shorter_side, center_crop, trivial_augment Image Norm: imagenet Max Images per Column: 2
**fusion_mlp**	Weight: 0.1 Hidden Sizes: 128 Activation: leaky_relu Drop Rate: 0.1 Normalization: layer_norm
**Optimization**	Optimizer: Adam W Learning Rate: 0.0001 Weight Decay: 0.001 LR Schedule: cosine_decay Max Epochs: 10 Gradient Clipping: 1 (norm) Loss Function: auto Focal Loss γ: 2.0
**LoRA**	Modules: query, value, ^*q*^, ^*v*^, ^*k*^, ^*o*^ Rank (r): 8 Alpha: 8
**Environment**	GPUs: 1 Batch Size: 16 (8 per GPU) Precision: 16-bit Workers: 2 Strategy: auto_select_gpus
**Backbone (electra)**	Architecture: ElectraForPreTraining Hidden Size: 768 Layers: 12 Heads: 12 Dropout: 0.1 Activation: gelu Vocab Size: 30522
